# ACG: rapid inference of population history from recombining nucleotide sequences

**DOI:** 10.1186/1471-2105-14-40

**Published:** 2013-02-05

**Authors:** Brendan D O'Fallon

**Affiliations:** 1 ARUP Labs, 500 Chipeta Way, Salt Lake City, UT, 84108, USA

**Keywords:** Coalescent, ARG, Ancestral recombination graph, Bayesian inference, Recombination

## Abstract

**Background:**

Reconstruction of population history from genetic data often requires Monte Carlo integration over the genealogy of the samples. Among tools that perform such computations, few are able to consider genetic histories including recombination events, precluding their use on most alignments of nuclear DNA. Explicit consideration of recombinations requires modeling the history of the sequences with an Ancestral Recombination Graph (ARG) in place of a simple tree, which presents significant computational challenges.

**Results:**

ACG is an extensible desktop application that uses a Bayesian Markov chain Monte Carlo procedure to estimate the posterior likelihood of an evolutionary model conditional on an alignment of genetic data. The ancestry of the sequences is represented by an ARG, which is estimated from the data with other model parameters. Importantly, ACG computes the full, Felsenstein likelihood of the ARG, not a pairwise or composite likelihood. Several strategies are used to speed computations, and ACG is roughly 100x faster than a similar, recombination-aware program.

**Conclusions:**

Modeling the ancestry of the sequences with an ARG allows ACG to estimate the evolutionary history of recombining nucleotide sequences. ACG can accurately estimate the posterior distribution of population parameters such as the (scaled) population size and recombination rate, as well as many aspects of the recombinant history, including the positions of recombination breakpoints, the distribution of time to most recent common ancestor along the sequence, and the non-recombining trees at individual sites. Multiple substitution models and population size models are provided. ACG also provides a richly informative graphical interface that allows users to view the evolution of model parameters and likelihoods in real time.

## Background

Reconstruction of population history from genetic data often requires computationally intensive Markov chain Monte Carlo strategies to estimate Bayesian posterior or likelihood surfaces (e.g. [1]). Tools that perform this task are sometimes called “genealogy samplers” [2] because they construct many quasi-independent samples of the genealogy describing the ancestry of the sequences. Genealogy samplers have become essential components of modern population genetic analysis, with the most popular tools, MrBayes [3,4], BEAST [5], IMa [6], and LAMARC [7] accumulating some 10,000 citations over the last decade. While these tools have rapidly increased in sophistication, with the exception of LAMARC they share a common limitation: they cannot be used to accurately analyze sequences with recombination. This restriction means that analyses cannot be carried out on most alignments of nuclear data, and as a result typical investigations are limited to mitochondria and non-recombining viruses. While LAMARC can be used on recombining sequences, analyses are impeded by slow performance and single runs may take several days or weeks to complete.

Several challenges face population genetics analysis with recombining data. When sequences recombine, their ancestry cannot be described by a simple tree or genealogy, and instead must be represented as an Ancestral Recombination Graph (ARG, [8]). All but the simplest of ARGs are difficult to visualize, and few resources are available for drawing, reading, and simulating ARGs. Additionally, the space of likely ARGs supported by a given alignment is often much larger than the space of trees, hence MCMC algorithms likely will take longer to converge. When satisfactory convergence is reached, few tools exist to extract meaningful information from ARGs.

Despite these challenges, the presence of recombination also facilitates some aspects of analysis. Because nearby sites may have partially independent histories more power may exist to infer population parameters. Similarly, evolutionary forces such as selection may create detectable genealogical features that would be obscured if all sites shared the same tree. For instance, a selective sweep may shorten the time to most recent common ancestor (TMRCA) in a given region, but such a shortening may be obscured if the region is completely linked to regions affected by different forces. Inference of complex demography may also be aided – for example, periods of admixture with other populations may cause some regions to have a relatively ancient TMRCA, but such features can only exist when recombinations allow some genomic regions to have an alternative history.

ACG (Analysis of reCombinant Genealogies) is a graphical desktop application that aims to overcome the challenges inherent in ARG inference, and to provide rapid and informative coalescent analysis of recombinant genetic data. It takes as input an alignment of homologous nucleotide sequences, and executes a Bayesian Markov chain Monte Carlo algorithm to infer the posterior distribution of parameters such as population size, recombination rate, transition to transversion ratio, as well as the history of the sequences represented by an ARG. Importantly, ACG computes the “full” likelihood of the ARG using a modified Felsenstein pruning algorithm [9], not an ad-hoc or composite likelihood. As with other genealogy samplers, the raw output of the program is a collection of parameters sampled from the Markov chain. ACG also contains many data collection tools and utilities designed to simplify the analysis of the sampled states.

## Implementation

Yang & Rannala [1] described a method for estimating the posterior distribution of a phylogenetic tree conditional on an alignment of genetic data using a Markov chain Monte Carlo (MCMC) approach. The method involves proposing small, successive changes in the structure of the tree and evaluating the likelihood that the tree produced the observed data using a Felsenstein pruning algorithm [9]. Proposed changes are accepted or rejected according to the Metropolis-Hastings criterion, which is an increasing function of the ratio of the likelihood of the proposed vs. current state. While originally proposed as a method of tree estimation, minor modifications of the algorithm allow for inference of additional model parameters, such as properties of the nucleotide substitution model, lineage birth and death rates (in the case of phylogenetic estimation), or population size (in the case of coalescent models). The basic approach described has experienced relatively wide adoption, with software tools such as MrBayes [4], LAMARC [7], and BEAST [5], applying, extending, and optimizing the algorithm. ACG continues to build upon this fundamental algorithm and, in a manner similar to the tools mentioned above, estimates the probability of an evolutionary model conditional on observation of an alignment of homologous nucleotide sequences. In mathematical terms, ACG produces an estimate of

(1) $$\mathrm{Pr}\left\{M/D\right\}=\mathrm{Pr}\left\{D/M\right\}\;\mathrm{Pr}\;\left\{M\right\}/\mathrm{Pr}\;\left\{D\right\}$$

where D is the input alignment and M is an evolutionary model containing parameters that are estimated from the data. At minimum, the model includes a description of how nucleotide sequences change over time (for instance, the Felsenstein 1984 or Timura-Nei 1993 model), a structure describing the ancestral relationships among the samples, and a function describing population size. Each of these sub-models may in turn encapsulate one or more parameters that are estimated from the data. For instance, the Timura-Nei 1993 model of nucleotide substitution involves two parameters that affect the transition-to-transversion ratio in addition to a vector describing stationary state nucleotide frequencies. As in LAMARC, the ancestry of the samples is represented by an Ancestral Recombination Graph (ARG), which is estimated from the data simultaneously with other model parameters.

To estimate the probability of the model given the data, ACG constructs and executes a Markov chain whose stationary state is the desired distribution (eq. 1). Generation of new states involves proposing a new value for a selected parameter, calculating the likelihood of the newly proposed state as well as the Hastings ratio associated with the proposal, and accepting or rejecting the state based on the Metropolis-Hastings-Green criterion [10,11]. A typical run involves repeating this procedure for some tens of millions of steps. States are sampled periodically and properties of model parameters are recorded by a variety of data collection utilities. If the chain has reached stationarity, the sampled parameters may be assumed to be correlated draws from the posterior density Pr{Model | Data}.

While the above scheme is similar to that used in other genealogy samplers, several aspects of the implementation are worthy of note. Most importantly, ACG implements data structures and MCMC proposal kernels that allow ARGs to be sampled from the data, where the probability of a particular ARG being sampled is proportional to its full likelihood under the data and some model of nucleotide substitution. Currently, the Felsenstein '84 (F84) and Timura-Nei '93 (TN93) substitution models are supported. Seven different proposal kernels operate on ARGs, these include some previously described and two novel kernels. ARG proposal kernels are detailed in the Additional file 1: Appendix A: ARG proposal kernels.

ARGs are a complex and rich source of information regarding the history of populations, and ACG provides several novel features that aid in interpreting the collection of ARGs sampled by the Markov chain. First, ACG by default tracks many of the bulk properties of ARGs, such as the number of recombination breakpoints and height of the deepest accessible nodes. In addition, ACG records the locations of all recombination breakpoints as well as the TMRCA across the sequence. ACG also provides utilities to examine the consensus tree at individual sites. These consensus trees are familiar, non-recombining trees ancestral to a single site only. Such trees may be useful when the ancestry in a small region is of particular interest, or when several such regions are to be compared. Further, ACG provides a companion utility (the *argutils* tool) that is capable of examining a single ARG and collecting information from it, such as the positions and heights of all recombination breakpoints, a list of all of the marginal trees contained in the ARG, or a plot of the TMRCA across the length of the sequence subtended by the ARG.

Users may interact with ACG in several ways. ACG features a simple command-line interface suitable for batch processing, but also provides a rich graphical user interface (GUI). The GUI allows users to both construct an analysis by selecting parameters, proposal kernels, and model priors, as well as to observe selected parameters and likelihoods as they change in real time as the chain progresses. Observed parameters and likelihoods can be viewed in trace or histogram form, and allow for rapid assessment of MCMC characteristics and convergence. Input files may also be saved and reloaded from within the GUI, and saved input can be executed from the command line.

A primary design goal of ACG is efficient computational performance and several techniques are used to speed calculations. First, every MCMC state involves calculation of new likelihoods followed by acceptance of rejection of the proposed state. If the state is accepted, the entire proposed proposed state must be moved to the current state before the next step can be initiated. In contrast to some other algorithms, ACG does not perform a full copy of the data and instead uses a reference-swapping technique to move the information to the desired location. Because the state data may be quite large and must be updated with every MCMC step, reference swapping results in a significant performance increase compared to copying. A second optimization technique involves identification of all identical alignment columns and computation of the data likelihood only once for each unique column. While many implementations involve some degree of alignment column ‘aliasing’, ACG performs this aliasing at every node where sites coalesce, again substantially reducing the number of likelihood calculations performed. Finally, ACG tracks which ARG nodes are affected by various proposals, and recomputes likelihoods only for the nodes and sites ranges affected.

## Results and discussion

### Resolution of individual recombination breakpoints in space and time

To demonstrate some of the unique features of ACG, we present a small analysis based on simulation data. To begin, an ARG was simulated with 10 sequences of length 10,000 sites under the standard neutral coalescent model with the population size parameter θ = 2*Nμ*= 0.02 and recombination parameter ρ = 2*Nr* = 1.0, using the *argutils* package included with ACG (where *N* is population size, μ is the mutation rate, and *r* is the recombination rate). The resulting ARG contained a total of 13 recombination breakpoints. All marginal trees were extracted again using *argutils*, and nucleotide sequences were simulated along each tree using *seq-gen*[12], using the F84 model of evolution. These sequences were then used as input to a standard ACG run. The run was conducted for 20,000,000 MCMC steps using a single chain, and completed in 53 minutes. Examination of parameter value traces suggested convergence in fewer than 1,000,000 MCMC steps for all parameters.

**Figure 1:**
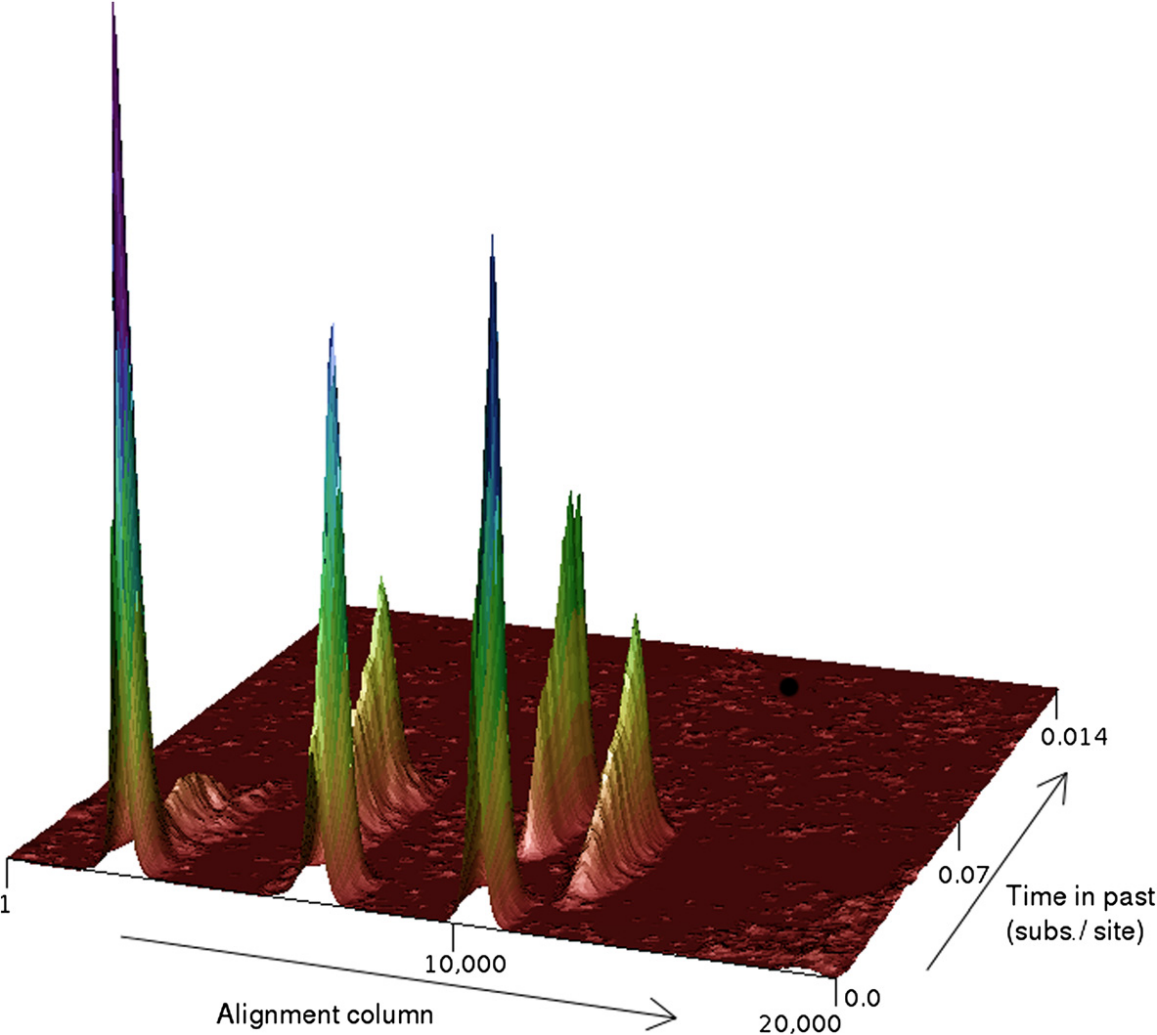
**Densities of recombination breakpoints sampled for a simulated alignment. **See text for details.

A novel feature of ACG is the ability to determine the locations of individual recombination breakpoints not only along the length of the sequence, but also in time. As ARGs are sampled from the running chain, recombination breakpoints are collected, and both the site of splitting as well as the height of the node containing the recombination are retained. These values are then added to a two-dimensional histogram, with one axis representing position along the sequence, and the other representing time. Figure 1 demonstrates that this method can yield an informative visualization of the locations of recombination breakpoints, with clusters of high density indicating that a high proportion of ARGs sampled bore a recombination near a particular location. The breadth and height of such clusters aid in quantifying the degree of uncertainty, with broad or shallow distributions indicating relatively low confidence. Because of the inclusion of the time dimension, this method can resolve multiple locations that occur at or near the same position on the sequence, but at different times, potentially aiding in the investigation of recombination hotspots where multiple recombinations might otherwise obscure one another.

**Figure 2:**
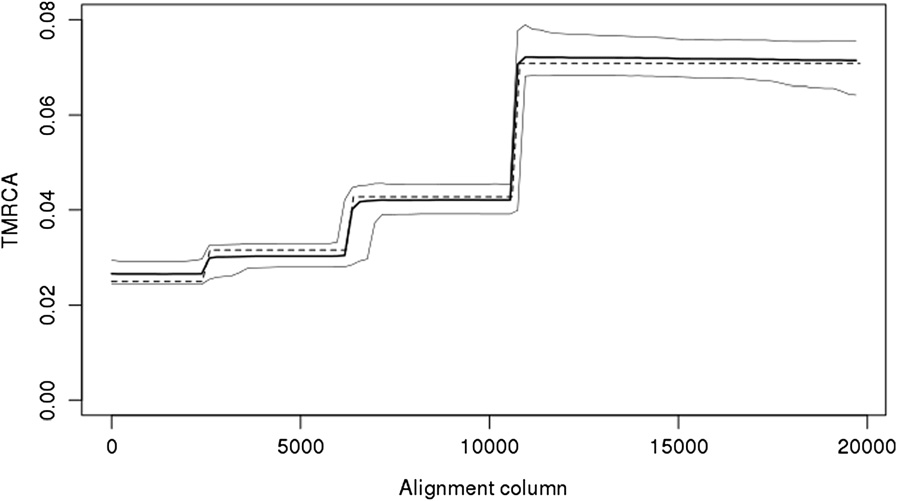
**Inferred time to most recent common ancestor (TMRCA) across the sequence for simulated sequence data.** Lighter colors indicate lower confidence. Dotted line indicates true TMRCA.

The presence of recombinations may also cause the TMRCA to vary across the length of the sequence. By sampling ARGs and assessing the TMRCA at multiple positions ACG can quantify how TMRCA changes across a sequence. Figure 2 demonstrates such a plot, indicating substantial variation in TMRCA in this data set, as well as very close correspondence in inferred TMRCA and the true TMRCA obtained from the simulated ARG.

**Figure 3:**
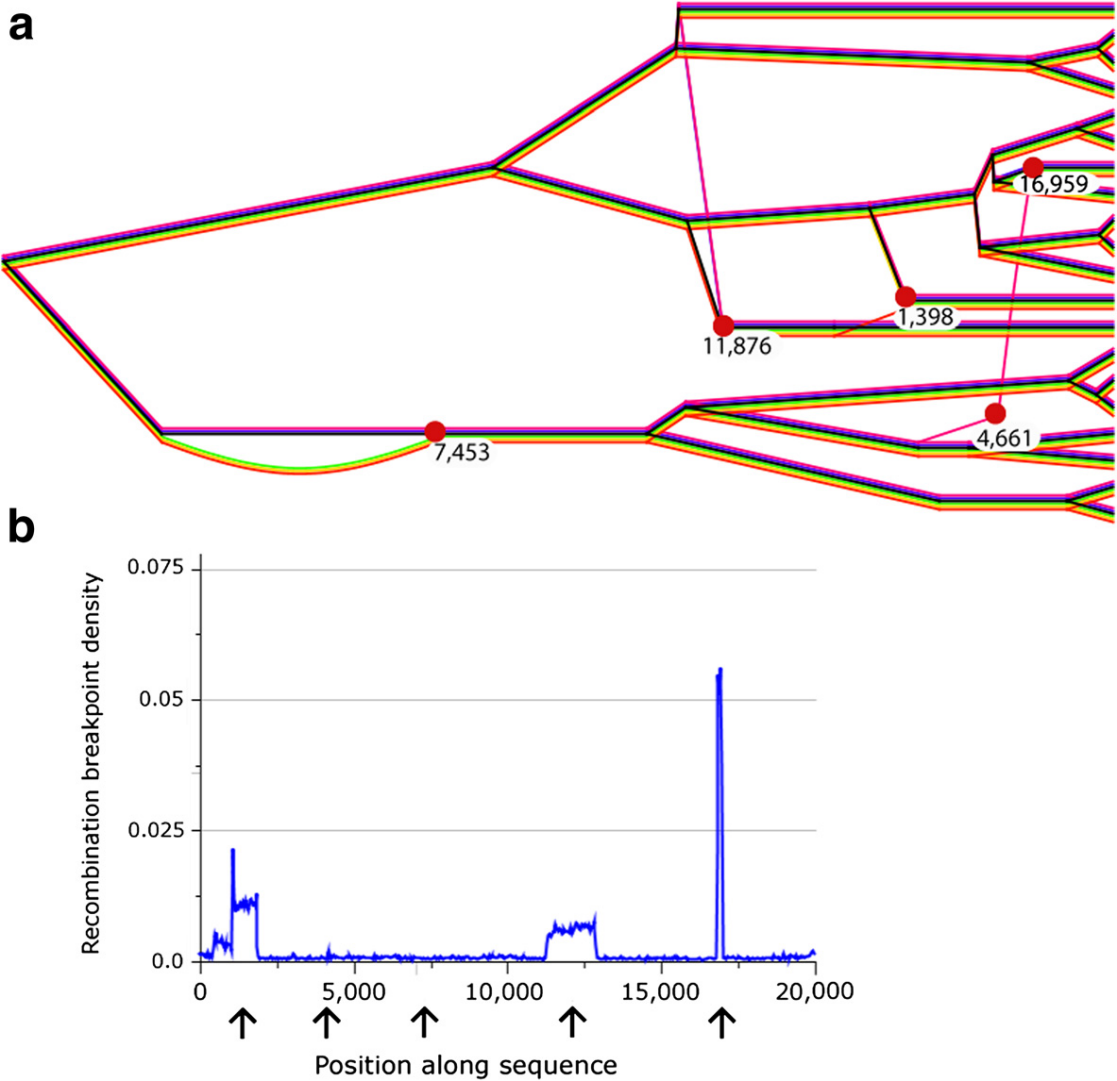
**a)Simulated ARG with all recombination breakpoints shown as red dots, with sequence position of the breakpoint indicated below. b)** Inferred positions of recombination breakpoints from sequence data simulated from ARG shown in a). Black arrows indicate true positions of recombination breakpoints.

To illustrate the ability of ACG to infer the sequence positions of recombination breakpoints, we created another dataset using the same procedure as above and collected the sequence positions of all recombinations, 5 in total. We again simulated sequence data on the ARG and used the sequence data as input to ACG. We conducted a single-chain ACG run for 10M steps, inferring the ARG, the transition-transversion ratio, population size and recombination rate from the data. Using the “Breakpoint Location Logger” the sequence positions of all inferred recombination breakpoints were collected (Figure 3b). ACG correctly identified three recombinations (at positions 1,398, 11,876, and 16,887), but found no evidence for the two remaining recombinations (at positions 4,661 and 7,453). Inspection of the ARG (Figure 3a) reveals that the two undetected recombinations each belong to a different class that are impossible to detect from sequence data alone. The branches created by the recombination at position 7,453 coalesce with one another before coalescing with any other branch, making the marginal trees on either side of the breakpoint identical. These types of recombinations are “trivial” in the sense that they have no impact on the topology of any tree in the ARG. Similarly, the recombination at position 4,661 does not affect any sites ancestral to the sampled sequences. The branch it affects is ancestral to another recombination at site 16,959, and the branch ‘contains’ only sites from 16,959-20,000. Thus, the split at position 4,661 does not affect any data in the sequences that were sampled.

### Empirical example

To demonstrate ACG’s utility on empirical alignments of recombining sequence data we investigate the history of a 10 Mb portion of the human X chromosome. The data set is composed of 12 X chromosome sequences from a worldwide sample of males obtained from the Complete Genomics Diversity panel, and hence is unambiguously phased (pseudo-autosomal regions were not examined). Summary information for the twelve individuals is listed in Table 1. The region spans chromosomal locations Xp11.4 – Xp11.23, and thus is located on the proximal region of the short arm. RefSeq annotations indicate 253 genes in the region investigated.

**Table 1:** Sample identifier and population of origin for samples used in the empirical example

**ID**	**Population**
NA07357	CEU (European ancestry, United States)
NA18501	YRI (Yoruba, Idaban, Nigeria)
NA18558	CHB (Han Chinese, Beijing)
NA18940	JPT (Japan, Tokyo)
NA19025	LWK (Luhya in Webuye, Kenya)
NA19649	MXL (Mexican ancestry, United States)
NA19670	MXL (Mexican ancestry, United States)
NA20510	TSI (Toscan, Italy)
NA20845	GIH (Gujarati Indian, India)
NA20846	GIH (Gujarati Indian, India)
NA20850	GIH (Gujarati Indian, India)
NA21737	MKK (Masai, Kinyawa, Kenya)

10 Mb is too large a region for a single run of ACG, hence the region was divided into 50 Kb fragments, with 10 Kb overlap between adjacent fragments, and ACG was run on each fragment independently. Initially, runs were conducted for 50,000,000 MCMC steps using 4 chains in a Metropolis-coupled scheme. The first 50% of steps were discarded as burn-in, and independence was assessed by comparing the means and standard deviations of the data likelihood trace between adjacent quartiles of the non-burn-in portion of the run. Some 20% of chains did not reach convergence in the initial run, for these chains ACG was run again using the maximally likely ARG found during the initial run as the starting ARG for the new run. This procedure was repeated until all chains reached convergence. Typical run time was 3–5 hours on a 2.2 GHz Intel Xeon quad-core processor.

**Figure 4:**
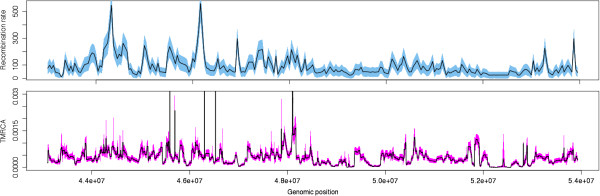
**Marginal TMRCA and recombination rate inferred over a 10Mb portion of the human X chromosome.** Shaded regions indicate 95% credibility region (TMRCA) and mean ± one standard deviation (recombination rate).

Figure 4 demonstrates the broad chromosomal features that can be assessed using ACG. For instance, the structure of the marginal TMRCA (the time to the most recent common ancestor at individual sites along the length of the sequence) is seen to vary widely, with tracts of relatively recent TMRCA (near site 52,000,000) and regions of very deep ancestry, in some cases extending beyond 0.003 subs./site. This type of analysis may have implications for the study of haplotype structure. For instance, regions of shallow TMRCA appear to be relatively broad, suggesting that fewer recombinations have occurred, and thus that haplotype blocks will likely extend over relatively long distances. Conversely, in areas with especially deep TMRCA the ancestry are likely to contain a greater total number of recombinations, reducing the length of haplotype blocks. Studies that utilize haplotype structure to infer recent selection (e.g. [13,14]), for instance, may benefit from this additional, relatively fine-grained source of information. Overall, both deep and shallow regions may indicate areas of evolutionary interest, such as ancient admixture or recent selective sweeps, respectively.

Figure 4 also demonstrates that ACG can be used to assess recombination rate and the number of recombination breakpoints in a specified region. Currently, ACG assumes that recombination rate is constant across sites and over time, although it was estimated independently for each 50Kb fragment. This functionality is similar to that provided by tools such as LDHat [15], but is likely to be more accurate than composite-likelihood based methods for several reasons. First, ACG considers the “full” likelihood of the ARG, not a composite of pairwise likelihoods. Additionally, ACG employs more flexible models of nucleotide substitution that co-estimate base frequencies and transition/transversion parameters along with other likelihood features. Finally, ACG provides not a point estimate of recombination rate but an estimate of the posterior distribution, allowing an appropriate characterization of the degree of uncertainty in the estimated parameter.

### Performance

A primary design goal of ACG is high computational performance. To compare the performance of ACG to that of the only other recombination aware genealogy sampler, LAMARC [7], 10 data sets each including 20 sequences of length 50Kb were generated using the following procedure. First, ARGs were simulated using the *argutils* package included with ACG with θ=0.01 and ρ=1. ARGs were decomposed into marginal trees also using *argutils*, from which nucleotide sequences were generated with *seq-gen*[12] using the F84 model of evolution. For each of the ten input alignments both ACG and LAMARC were run for exactly one hour of real time, and both tools estimated θ, ρ, the transition- to-transversion ratio, as well as the structure of the ARG. Log files of parameter values and the likelihood of the data conditional on the ARG were produced for all runs with sampling every 5000 steps from ACG and every 20 steps from LAMARC. The program Tracer (v 1.5) was then used to examine the log files and calculate the effective sample size (ESS). To assess convergence we examined the likelihood of the data conditional on the ARG (the “data likelihood”).

In several respects ACG significantly outperformed LAMARC. In raw number of MCMC states processed ACG was over 100-fold faster than LAMARC, computing on average 1.8×10^7^ total states, while LAMARC computed 1.4×10^5^. In terms of effective sample size (ESS) for the data likelihood, the mean for ACG was 1284 (range 178–3122) indicating satisfactory convergence, while for LAMARC the mean was 23 (range 6–53). Similarly, ESSs for the scaled population size parameter θ were on average 1861 (range 188–3964) for ACG and 19 (range 8–36) for LAMARC. In addition, ACG's memory requirements are typically modest, and 512MB per chain is sufficient for typical data sets. Finally, we note that these results used only a single chain. Because ACG can execute multiple heated chains simultaneously the performance margin over LAMARC is likely to be further increased when multiple CPU cores are available.

### Validation

**Figure 5:**
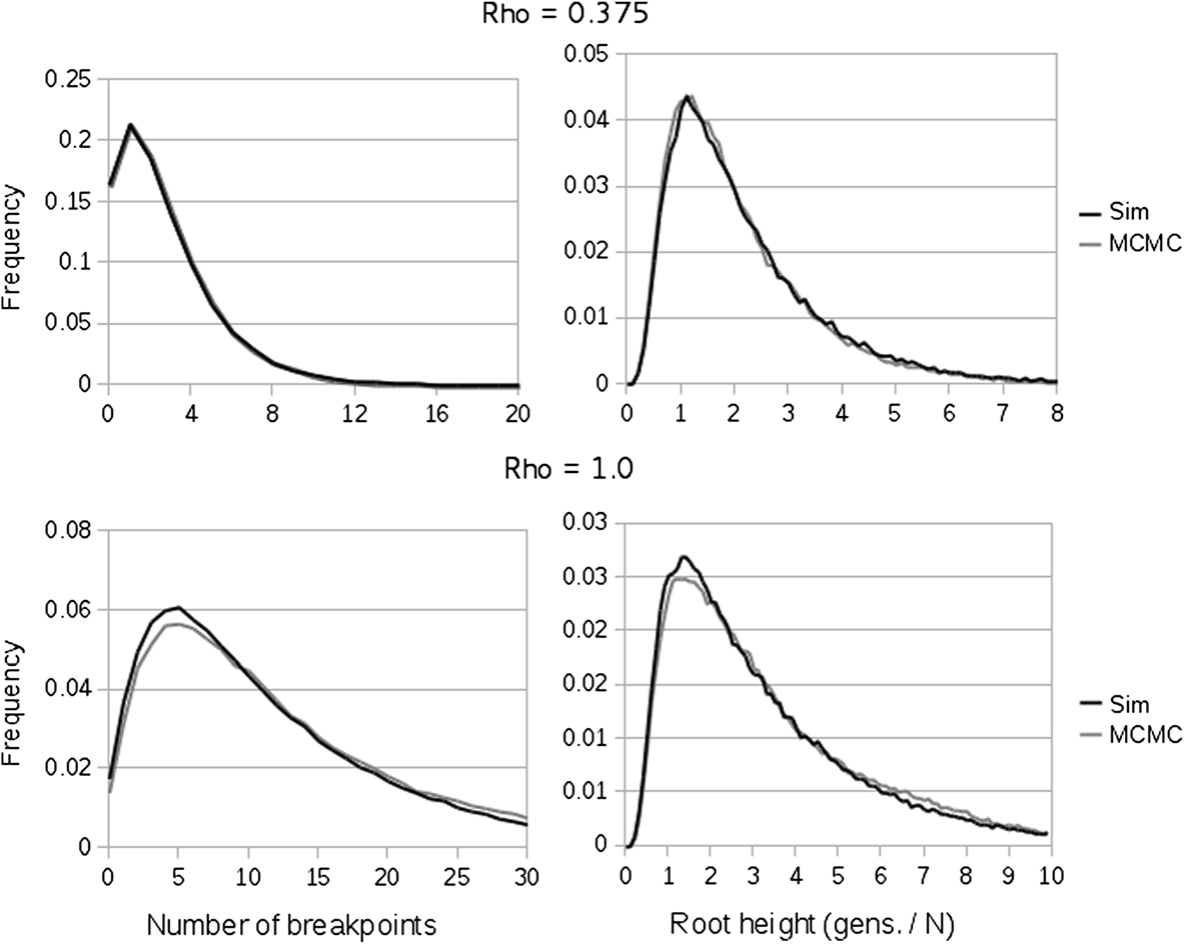
Distributions of the number of recombination breakpoints (left column) and root height (right column) obtained from direct backward simulation of ARGs (black lines) and from ARGs sampled from the MCMC implemented in ACG (gray lines).

Algorithms with the complexity and sensitivity of ACG are prone to error. Here, we present several analyses demonstrating that ACG performs as expected. Perhaps the most sensitive component of the analysis is ensuring that the distribution of ARGs sampled in the absence of input data matches the theoretical distribution described by neutral coalescent theory. To assess the properties of ARGs sampled by ACG in the absence of data to those generated by neutral simulation, we held population size constant at 1.0 and ran ACG for 2.5×10^7^ MCMC steps, recording the height of the root node every 1000 steps. Figure 5 demonstrates that ARGs sampled from the MCMC are very similar to those generated under direct (backward) simulation in terms of both the distribution of the number of recombination breakpoints as well as the distribution of height of the deepest node.

**Figure 6:**
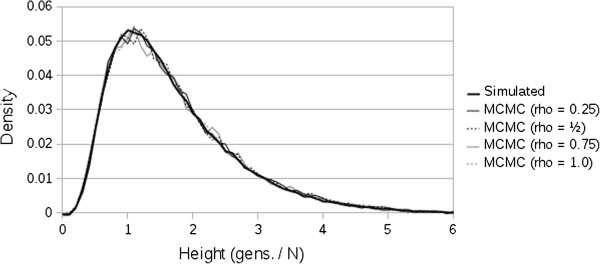
Distribution of time to most recent common ancestor at a single site, estimated from direct backward simulation of coalescent trees (thick black line), and the MCMC scheme implemented in ACG for several recombination rates (gray lines).

As an additional test of ARG correctness we note that the distribution of non-recombining trees sampled from a single site should be identical to standard neutral coalescent trees, because the presence of recombinations elsewhere along the sequence should not affect any property of trees sampled. We therefore examined the distribution of TMRCA at trees sampled from a single site by the MCMC, for varying levels of recombination, and compared these distributions to those obtained from direct backward simulation. The distributions show close correspondence, and no deviation associated with increasing levels of recombination (Figure 6) is apparent.

Another potential source of error stems from the accuracy of the likelihood computations, in particular the likelihood of the data conditional on the ARG (the “data likelihood”). To ensure that ACG computes the correct likelihood at every MCMC step, test classes were implemented that emit marginal trees and their associated likelihoods periodically during a run. Utility scripts were developed to read these files and input each tree into an external program capable of computing the likelihood of an alignment given a tree, in this case the DNAMLK tool distributed with the phylip (3.69) package [16]. When ACG functions as expected, the sum of the log likelihoods for each marginal tree should be equal to the full log likelihood of the ARG. This procedure has been conducted numerous times during development and has been used to identify and correct data likelihood calculation errors.

## Conclusions

Bayesian genealogy samplers provide a means of inferring a wide variety of population parameters from genetic data. ACG builds upon techniques developed in earlier samplers, and significantly extends the range of input data that can be considered as well as the types of data that can be collected. Most importantly, ACG’s use of an ARG to represent the ancestry of the sequences enables the examination of alignments of nuclear DNA, opening new avenues of investigation for common alignments of sequence data (although the data must be properly phased). While ARGs present considerable inferential challenges compared to non-recombining trees, ACG provides several utilities for ameliorating these difficulties and extracting useful information from the cloud of ARGs sampled. These utilities include estimation of TMRCA along the sequence, locations of recombination breakpoints in space and time, and construction of consensus trees at particular sites of interest. In addition to these tools, the *argutils* utility included with ACG provides a number of convenient functions, including breaking an ARG into marginal trees, extracting a single tree from a specific site, enumerating recombination positions from an ARG, and simulating neutral ARGs.

While ACG shares some features with LAMARC, including the use of an ARG to represent ancestral relationships, several important features distinguish the programs. As demonstrated above, ACG is roughly 100-fold faster than LAMARC. Additionally, ACG can estimate the shapes of marginal trees at specific sites, the locations of recombination breakpoints along the sequence as well as in time, and the time to most recent common ancestor along the sequence. ACG can also import data from the. VCF file format commonly used in next-generation sequencing projects. LAMARC, in contrast, has several features ACG does not, most significantly the ability to model multiple populations and the migration rates between them.

Finally, ACG offers a convenient graphical interface that allows users to not only construct an analysis, but also to observe the evolution of various parameters and likelihoods in trace or histogram form in real time, allowing researchers to monitor many features of the analysis as it unfolds.

## Availability and requirements

ACG is freely available for academic use and will operate on any platform with a Java Virtual Machine version 1.6 or higher installed.

Project name: ACG

Homepage: http://arup.utah.edu/acg

Operating systems: Platform independent

Programming language: Java (v 1.6)

Requirements: Java 1.6 or higher

License: Copyright 2012 Brendan O’Fallon, freely available for academic use

## Abbreviation

ARG: Ancestral Recombination Graph.

## Competing interests

The author declares that he have no competing interests.

## Supplementary Material

Additional file 1 **Appendix A.** ARG proposal kernels. Click here for file
