# Supplementary material for: ACG: rapid inference of population history from recombining nucleotide sequences
Source: BMC Bioinformatics. 2013 Feb 5;14:40. doi: 10.1186/1471-2105-14-40 (PMC3575405; doi:10.1186/1471-2105-14-40)
Supplement: Additional file 1 — Appendix A. ARG proposal kernels. [file 1471-2105-14-40-S1.pdf]

Appendix A : ARG proposal kernels

Described below are the proposal kernels that have been implemented in ACG that operate on Ancestral Recombination Graphs (ARGs). In ACG, ARGs are directed graphs composed of three distinct node types and augmented with additional information regarding the nucleotide sites subtended by the graph. In general, an ARG may extend from sites *m .. n,* where $l = n - m$ is the total number of sites subtended. The three nodes type are tips, coalescent nodes, and recombination nodes. Tip nodes contain exactly one parent and no offspring. Coalescent nodes contain exactly two children and one parent. Exactly one coalescent node, the "root" node, has zero parents. Recombination nodes contain two parents and one offspring. Henceforth, let t-node denote a tip node, c-node denote a coalescent node and r-node denote a recombination node. R-nodes also contain a single integer describing the site at which the recombination occurs. Recombinations always occur between two sites, thus the breakpoint actually contains the index of the highest-valued site that is not greater than the recombination. Using this description recombinations may occur at sites *m .. n-1.* The two parents of a recombination in question, say p_0 and p_1, are associated with a range of sites defined by the breakpoints. Specifically, if the breakpoint is at site *r,* sites in the half-open interval [n..r) 'lead to' p_0, and sites in [r, m) 'lead to' p_1 . Finally, all nodes are assigned a 'height', which describes the distance of all nodes from the tips of the graph. In the current implementation, all tip nodes have height = 0, although this is not required in general.

We further restrict ARGs to satisfy the following conditions.

1. The node with the greatest height in the graph is a c-node, which we label the 'root', which is the only node with zero parent nodes.
2. At no height *h* is the number of branches crossing *h* = 1.

Below we describe in detail each proposal kernel. The default relative sampling frequency of each kernel is listed after the name.

1. *Node height modifier* (frequency 10.0)

The node height modifier alters the distance of a single coalescent or recombination node from the tips. A single, non-root c-node or r-node is selected with equal probability, and the minimum height of its parent(s) and maximum height of its offspring is recorded. A new uniformly distributed random number between the two heights is generated, and the proposed node height is assigned to that value.

2. *Root height modifier* (frequency 2.0)

This modifier proposes a new value for the height of the root node, and depends on an exogenously defined variable, say *m*. Given the current root height, say *r,* a new root height is proposed as a uniformly defined random variable in the interval (r-m/2, r+m/2). If the newly proposed root height is less than the maximum height of the offspring, the proposed height is reflected over this offspring height.

3. *Breakpoint shift* (frequency 1.0)
This modifier alters the location of a single recombination breakpoint in a sliding-window fashion. First, a single r-node is selected with equal probability from among all r-nodes. Given some window size, say m, and the current position, say p, the new position is a uniformly distributed variable in $(p\text{-}m/2, p+m/2)$. As with the root height modifier, proposals beyond the boundary (in this case, beyond the sites over which the ARG is defined) are reflected over the boundary. The window size m is changed during execution to maintain a proposal acceptance rate near 25%.

4. *Breakpoint swap* (frequency 1.0)
This modifier swaps the two parents of a recombination node. First, a single r-node is selected with equal probability from among all r-nodes. Then, using the terminology described above, p_0 and p_1 are exchanged such that sites $[n..r)$ 'lead to' p_1, and sites in $[r, m)$ 'lead to' p_0.

5. *Local swap* (frequency 2.0)
This modifier changes the branching structure of the ARG by moving one offspring or parent node to nearby branch. First, an non-tip node is selected with equal probability from among all non-tip nodes. If the node is a c-node, the offspring with the greatest height is selected to move. The offspring node is then transferred to the branch connecting the c-node to the other offspring, as in figure A, below. If the chosen node is an r-node, then the parent of the r-node with the smallest height is moved to the branch connecting the r-node to the other parent. No node heights are altered during any step.

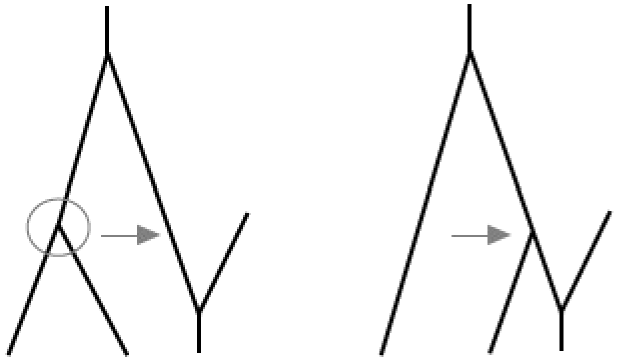

Figure A: Diagram of local swap move performed on the offspring of a c-node.

6. *Wide Swap* (frequency 1.0)

This procedure is similar to the local swap, but introduces more radical changes to the branching structure. In a manner similar to the local swap, an internal node is chosen uniformly from among all internal nodes. If the internal node is a c-node, then the offspring with the greatest height is chosen to move. If the internal node is an r-node, then the parent with the smallest height is chosen to move. Potential recipient branches for the moving node are then collected from among all branches that cross the height of the moving node (black dots, Figure B, below). A recipient location is chosen uniformly from among these potential locations, and the moving node is attached to this location. As in the local swap move, no node heights are altered.

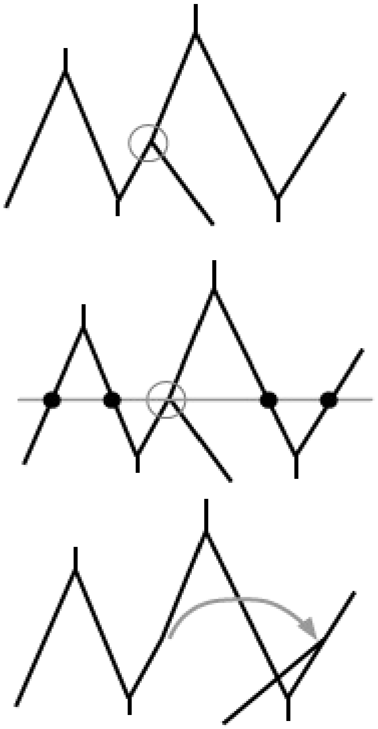

Figure B: Diagram of 'wide swap' proposal kernel. Top: a node is chosen to move by selecting an internal node in a manner similar to the local swap procedure. Middle: potential recipient locations are found on all branches that cross the height of the moving node. Bottom: a recipient location is chosen and the moving node is attached to it.

7. *Add/ remove branch*: (frequency 2.0)
This kernel either adds or removes one branch, consisting of a recombination node and a coalescent node.
Adding branch proposal: Heights for the new nodes are chosen in the following manner. First, a truncated exponential random variate is generated with mean $r/2$ and maximum r, where r is the height of the root node of the ARG. Second, a height for the coalescent node is chosen by generating another exponential random variate with rate $r/2$ (but no maximum), and adding to this the height of the first node. Next, potential branches on which the new nodes will reside are collected by examining all branches that cross the heights generated in the previous step.

If the height of the new c-node is greater than *r*, the c-node will be the new root and an additional branch is generated to connect the previous root to the new root. The new branch is inserted into the ARG at the chosen locations. Finally, a location for the new recombination breakpoint is chosen uniformly among all sites at which recombinations may occur.

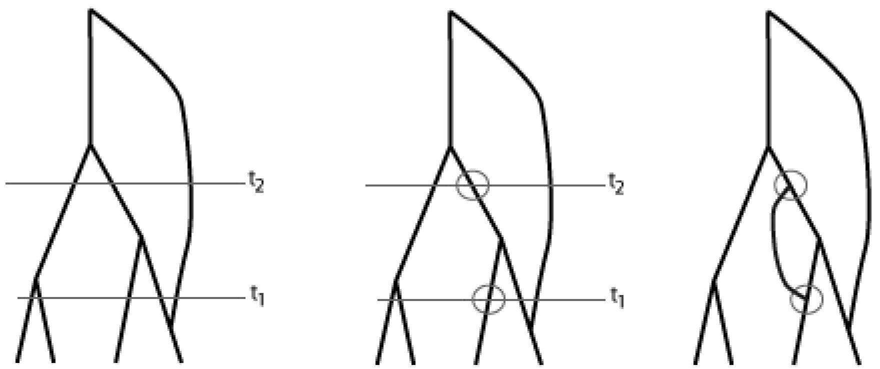

Figure C: Procedure for adding a new branch to an ARG. Left: two random heights are selected. Middle: Recipient locations are chosen for branches that cross the heights. Right: a new branch is added that connects the two chosen recipient locations.

Branch removal: All 'removable' branches are collected in the following manner. A branch is defined as an r-node and a c-node, where the c-node is necessarily a parent of the r-node. A removable branch is branch whose removal will not create an ARG with only a single branch passing through any given time. A branch is selected at uniform from among all removable branches, and is then removed from the ARG.

Unlike other ARG proposal mechanisms, both the add and remove procedures generate nontrivial Hastings-Green ratios. The probability of a given add move is the following:

$$\lambda e^{-\lambda x} \quad \lambda e^{-\lambda(y-x)} / (1-e^{-\lambda})$$

where λ is half the height of the root node, *x* is the height of the new r-node, *y* is the height of the new c-node. The probability of a remove-branch move is the reciprocal of the number of removable branches, which is tabulated on each add and remove step. Finally, following the reversible-jump methodology described in Robert & Casella (2010), a Jacobian term of 2 is induced on the add move, and 1/2 on the reverse move.

Robert, C. P., Casella, G. 2010. *Monte Carlo Statistical Methods* (2nd edition). Springer Verlag NY
